# Non-imprinted allele-specific DNA methylation on human autosomes

**DOI:** 10.1186/gb-2009-10-12-r138

**Published:** 2009-12-03

**Authors:** Yingying Zhang, Christian Rohde, Richard Reinhardt, Claudia Voelcker-Rehage, Albert Jeltsch

**Affiliations:** 1 School of Engineering and Science, Jacobs University Bremen, Campus Ring 1, D-28759 Bremen, Germany; 2 Max Planck Institute for Molecular Genetics, Ihnestrasse 63-73, D-14195 Berlin-Dahlem, Germany; 3 Jacobs Center on Lifelong Learning and Institutional Development, Jacobs University Bremen, Campus Ring 1, D-28759 Bremen, Germany

## Abstract

**Background:**

Differential DNA methylation between alleles is well established in imprinted genes and the X chromosomes in females but has rarely been reported at non-imprinted loci on autosomes.

**Results:**

We studied DNA methylation of cytosine-guanine dinucleotide (CpG) islands on chromosome 21 in leukocytes from several healthy individuals and observed novel cases of pronounced differential methylation of alleles. Allele-specific methylation affected complete CpG islands with methylation differences between alleles of up to 85%. The methylation differences between alleles were strongly correlated with the genotypes, excluding a connection to imprinting. We show that allele-specific methylation can lead to allelic repression of the methylated gene copy. Based on our results, allele-specific methylation is likely to affect about 10% of all human genes and to contribute to allele-specific expression and monoallelic gene silencing. Therefore, allele-specific methylation represents an epigenetic pathway of how genetic polymorphisms may lead to phenotypic variability. In most cases, we observed that some, but not all, heterozygous individuals showed allele-specific methylation, suggesting that allele-specific methylation is the outcome of an epigenetic drift, the direction of which is determined by the genetic differences between the alleles. We could show that the tendency to acquire hypermethylation in one allele was inherited.

**Conclusions:**

We observed that larger differences in methylation levels between individuals were often coupled to allele-specific methylation and genetic polymorphisms, suggesting that the inter-individual variability of DNA methylation is strongly influenced by genetic differences. Therefore, genetic differences must be taken into account in future comparative DNA methylation studies.

## Abstract 2

Single-nucleotide polymorphisms located within CpG islands are found to cause differences in CpG methylation between alleles, reflecting differences in gene expression.

## Background

DNA methylation is a major epigenetic process that plays essential roles in gene expression regulation, development and disease [1-3]. In mammals, differential DNA methylation between alleles occurs in imprinted genes [4,5] and on the female X chromosomes [6,7]. So far, there have been few reports about allele-specific methylation (ASM) on autosomes not connected to the parental inheritance of the alleles [8-10]. In imprinting and X-chromosome inactivation, ASM leads to monoallelic expression of genes, and in some cases of non-imprinted, autosomal ASM, a correlation between DNA methylation and allele-specific gene expression has been documented as well [8,11].

In a previous work, we discovered ASM of three cytosine-guanine dinucleotide (CpG)-rich regions in gene promoters in leukocyte DNA derived from a healthy individual using bisulfite conversion, subcloning and sequencing [12]. Because of the important potential contribution of ASM to gene expression and disease [13], we initiated a larger survey to find more examples of ASM and to understand if the ASM of these regions relates to gene imprinting or is sequence-dependent. Therefore, we studied the methylation pattern of 16 CpG-rich regions in gene promoters of chromosome 21 in up to 38 individuals by bisulfite conversion, subcloning and sequencing of individual clones. Additionally, we checked the inter-individual DNA methylation difference at these gene promoters with respect to a potential link to age and gender differences.

## Results

Based on our previous work on DNA methylation analysis of gene promoters on chromosome 21 in blood derived from one individual [12], we selected 6 low methylated (methylation <30%), 7 intermediately methylated (methylation between 30 and 70%) and 3 highly methylated (methylation >70%) amplicons and studied the DNA methylation status of them in the blood derived from 10 old and 10 young individuals (5 males and 5 females in each group). The three amplicons (176_1, 176_2 and 23_2) that previously showed ASM were included in the analysis. Detailed information on the amplicons and individuals is shown in Table 1 and Additional files 1 and 2. The amplicons are all located in CpG-rich regions surrounding the transcriptional start sites of genes.

**Table 1:** Allele-specific methylation of amplicons among individuals and correlation with genotype

	Genes and amplicons					
	
	C21orf81		*HSF2BP*	*RIPK4*	*CBR1*	
			
	23_1	23_2	262	232	176_1	176_2
Length (bp)	370	426	400	241	302	476
CpG sites	31	26	42	25	31	44
SNP (strand) dbsnp build 129	A/C (-) rs2297246	A/C (+) rs56270809	A/G (-) rs2838343	A/G (+) rs55860816	del/no del (+) rs41563015	C/G (+) rs25678
Individuals studied	38	19	33	36	21	21
Individuals with SNP	17	8	20	11	1	8
Individuals with SNP and ASM	8	7	19	3	1	1
Coupling of ASM and genotype	Always: C hyper- methylated	Always: C hyper- methylated	18 cases: G hyper- methylated in right part of amplicon	Always: G hyper- methylated	Always: del hyper- methylated	Always: C hyper- methylated
			1 case: A hyper- methylated in left part of amplicon			
Average allelic methylation difference in ASM cases	53%	60%	54% (G hyper- methylated), 64% (A hyper- methylated)	39%	50%	60%
Individuals with SNP but without ASM	9	1	1	8	0	7
Individuals without SNP	21	11	13	25	20	13

ASM, allele-specific methylation; CpG, cytosine-guanine dinucleotide; SNP, single nucleotide polymorphism.

### Inter-individual variation of DNA methylation

**Figure 1:**
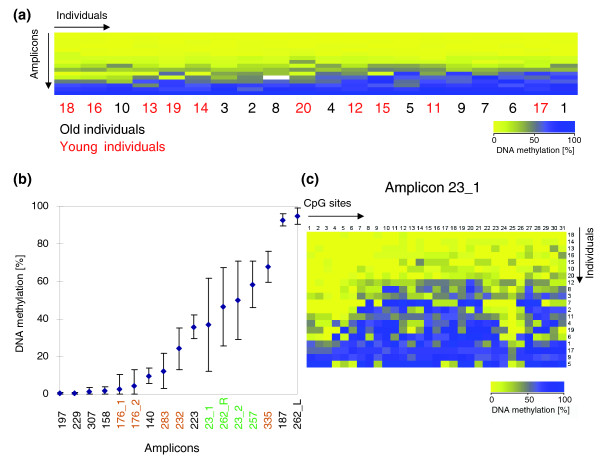
Inter-individual variation of DNA methylation. (a) DNA methylation levels of all amplicons studied in blood from ten old (1 to 10) and ten young (11 to 20) individuals. The data were sorted by the average level of DNA methylation among amplicons and individuals. Old individuals are represented by black numbers, young individuals by red numbers. The continuous yellow to blue color code represents the DNA methylation level of the PCR products. (b) Average DNA methylation level of all 16 amplicons among 20 individuals (1 to 20). The error bars indicate the standard deviation among the samples. Amplicon 262 was split into 262_L (left part) and 262_R (right part) because of the different methylation observed in these two parts. Green color labeled amplicons showed a maximal methylation difference between individuals of more than 50%; orange labels indicate amplicons with >30% methylation difference. (c) DNA methylation pattern of amplicon 23_1 in individuals 1 to 20. Each column represents a single CpG site. Each row corresponds to one individual.

A summary of the methylation of all amplicons in different individuals is shown in Figure 1a. The methylation levels of highly methylated or completely unmethylated amplicons were usually similar among individuals, while the intermediately methylated amplicons showed higher variability (Figure 1b). Four amplicons showed a maximal inter-individual difference in their methylation levels of >50% and another five amplicons showed variances of >30%. As an example, the methylation pattern of amplicon 23_1 in 20 individuals is shown in Figure 1c. When comparing the methylation difference among individuals, we did not observe any significant methylation difference between old and young individuals or males and females. This is consistent with former results where no significant correlation of methylation changes with gender or age was detected [14].

We observed that amplicons with a high methylation variance among individuals often showed a biphasic distribution of methylation comprising clones that were mainly unmethylated or highly methylated. This led to the question of whether the DNA methylation varied from one cell to another or between the two alleles in each cell. In some of these cases, the alleles could be differentiated by the presence of a single nucleotide polymorphism (SNP) in the sequenced region such that the sequencing reads could be sorted according to the allelic origin. In many of these cases we observed a clear correlation of genotype and methylation levels, indicating the presence of ASM (Table 1). To exclude that clonal PCR after bisulfite conversion might have caused these results, we performed individual bisulfite genomic sequencing analysis twice for cases where ASM was observed and always obtained similar results (Additional file 3). In addition, we used digestion with methylation-sensitive restriction enzymes to confirm the ASM (Additional file 3). For amplicons with ASM, we included more individuals in the analysis.

### Allele-specific methylation and expression of the first exon of the C21orf81 gene

**Figure 2:**
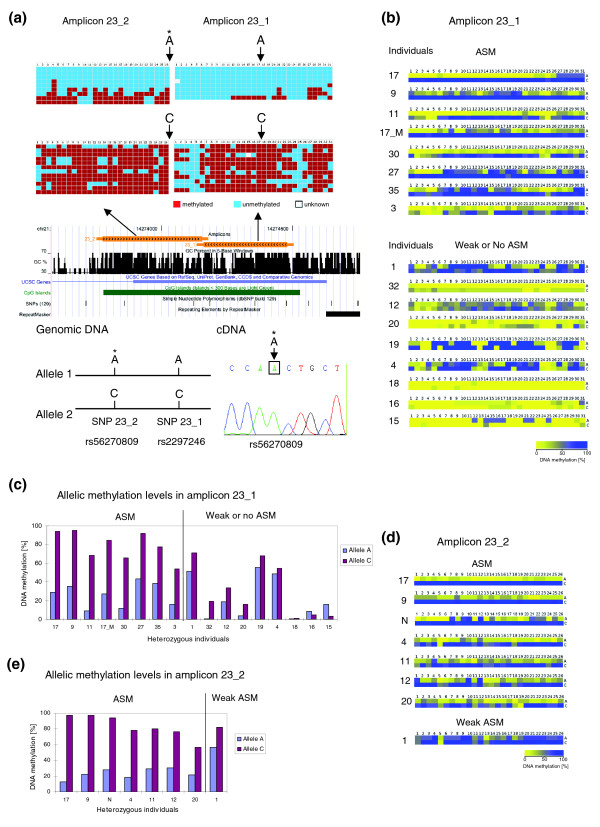
Allele-specific methylation and expression of amplicons 23_1 and 23_2. (a) Example of ASM of amplicons 23_1 and 23_2 in the first exon of gene C21orf81. The figure shows the position of these two amplicons in the UCSC genome browser and the methylation pattern of alleles in both amplicons. Each row corresponds to one clone of bisulfite PCR products. Each column corresponds to one CpG site in the studied region. The color code indicates different methylation states of the CpG sites (blue, unmethylated; red, methylated; white, methylation state unknown). The clones in each amplicon were sorted according to their genotype at two SNPs (dbsnp rs56270809 on 23_2 and dbsnp rs2297246 on 23_1), the positions of which are indicated by an arrow. By sequencing of the genomic DNA we identified the haplotype of all samples with ASM to be either A/A or C/C as schematically shown in the lower part of the figure. Expression analysis showed that the gene is exclusively expressed from the unmethylated A allele (indicated by an asterisk) in leukocytes (data are shown from individual 11). (b) Compilation of results from all heterozygous individuals in amplicon 23_1. The individuals were sorted according to the decreasing methylation difference between alleles. Each row corresponds to a single allele from one individual. Each square represents the average methylation of a CpG site encoded in a continuous yellow to blue color code. The original methylation patterns of all samples with ASM are shown in Additional file 4. (c) Methylation level of each allele from all heterozygous individuals in amplicon 23_1. (d) Compilation of results from all heterozygous individuals in amplicon 23_2. The original methylation patterns of all samples with ASM are shown in Additional file 4. (e) Methylation level of each allele from all heterozygous individuals in amplicon 23_2.

We observed strong ASM on the first exon of the C21orf81 gene where we studied the methylation pattern of amplicon 23_2 and 23_1, which are located next to each other and cover almost the entire CpG island (Figure 2a). The analysis for amplicon 23_1 was performed in 38 individuals, 17 of which had an A/C SNP in the sequenced region. Out of them, we observed ASM with a methylation difference of >30% in eight cases, always having the A allele hypomethylated (Table 1; Figure 2; Additional file 4). Some individuals showed a weaker DNA methylation difference between alleles, and in other cases no DNA methylation difference was detectable. Within the 21 homozygous individuals, we observed a biphasic distribution of DNA methylation levels in 9 cases, suggesting the potential presence of ASM, although this could not be identified because of the lack of a characteristic SNP.

The methylation pattern of amplicon 23_2 was studied in 19 individuals, including one (called 'N' in Figure 2 and the following) previously analyzed [12]. Altogether, eight individuals had an A/C SNP in this region and seven of them showed strong ASM (Table 1; Figure 2d; Additional file 4). In all ASM cases, the A allele was less methylated than the C allele (Figure 2e). For the 11 homozygous individuals, biphasic DNA methylation was observed in 7 cases.

We identified the correlation between the SNPs in amplicon 23_1 and 23_2 in the genomic DNA sequence of all individuals with ASM. When both SNPs were present, we always observed A-A and C-C haplotypes. In three such cases (individuals 9, 11 and 17), the A alleles in both amplicons were hypomethylated, indicating that pronounced ASM can span a whole CpG island (Figure 2a; Additional file 4). In individuals 3 and N, ASM was observed in one amplicon (either 23_1 or 23_2) and the other amplicon showed biphasic methylation, but did not contain the SNP, suggesting that the whole CpG island has ASM as well in these cases. However, there were also cases in which the methylation states of alleles in these two amplicons were not coupled. In individuals 4, 12 and 20, ASM was observed in 23_2, but there was no obvious methylation difference between alleles in amplicon 23_1. No methylation difference between alleles in both amplicons was observed in one individual (individual 1).

To determine if ASM leads to monoallelic expression of the C21orf81 gene, we prepared RNA from leukocytes of individuals 9 and 11, which showed strong ASM of both amplicons, and converted it into cDNA. Sequencing of the cDNA revealed that the gene was exclusively expressed from the unmethylated A allele, indicating the ASM correlates with allele-specific gene expression (Figure 2a).

We were able to check the methylation pattern of amplicon 23_1 in the parents of individual 17 (male). Like individual 17, the mother (17_M) had an A/C SNP with the C allele methylated. The father (17_P) had an A/A genotype and showed slight biphasic methylation but did not show clones with high methylation characteristic for C alleles (Additional file 4).

### Allele-specific methylation of a CpG island in the *HSF2BP* gene

**Figure 3:**
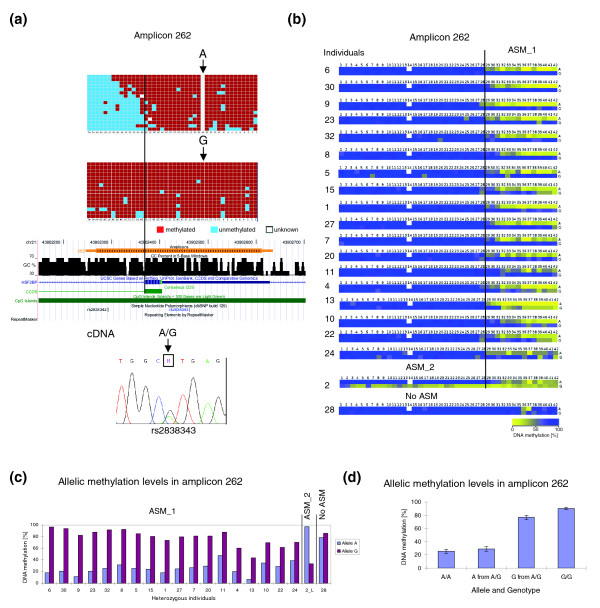
ASM of amplicon 262. (a) Example of ASM of amplicon 262. The figure shows the position of the amplicon in the UCSC genome browser, the positions of the SNP (dbsnp rs2838343) used to discriminate alleles, and the allelic methylation, basically as described in the legend to Figure 2a. The line indicates the border between the methylated and unmethylated part of the amplicon. Sequencing of cDNA showed that the gene is biallelically expressed (data are shown from individual 10). (b) Compilation of results from all heterozygous individuals. Two patterns of ASM were observed in amplicon 262. The line indicates the border between the methylated and unmethylated parts of the amplicon. For further details see the legend to Figure 2b. The original methylation patterns of all samples with ASM are shown in Additional file 5. (c) Methylation level of each allele from all heterozygous individuals. For this analysis the amplicon was split into two pieces corresponding to its left part (28 CpG sites) or right part (14 CpG sites). The data labeled with ASM_1 refer to the right part of the amplicon, ASM_2 refers to the left part and no ASM refers to the full amplicon. (d) Methylation levels of homozygous individuals with A/A or G/G genotypes compared with the methylation levels of A or G alleles from heterozygous individuals. In this analysis the results obtained for individual 2_L were included. The error bars indicate the standard rrror of the mean. After omission of 2_L, the methylation levels of the A and G alleles from A/G heterozygous individuals were 28% and 79%, respectively.

We also identified ASM on amplicon 262 containing 42 CpG sites, which is located on a CpG island in the *HSF2BP *gene (Figure 3a). The methylation state of this region was studied in 33 individuals. In many cases, a clear boundary between methylated CpG sites and unmethylated CpG sites was observed in the same amplicon (Figure 3a, b). Out of 20 individuals with an A/G SNP, ASM was observed in 19 cases (Table 1; Figure 3; Additional file 5), which showed two different patterns of ASM (Figure 3b). In 18 individuals, the first 28 CpG sites were methylated in both alleles, whereas in the remaining part of the amplicon, the A allele was hypomethylated. In one case (individual 2), the G allele was hypomethylated throughout the entire amplicon, whereas the A allele was methylated in the first 28 CpG sites but low methylated on the remaining 14 CpG sites similar to what was observed for the A allele in the other individuals. Absence of ASM was only observed in one heterozygous individual (individual 28). In this case, both alleles were highly methylated throughout the whole amplicon (Figure 3b). The methylation difference between alleles of all heterozygous individuals in amplicon 262 is summarized in Figure 3c. We analyzed the expression state of the alleles in leukocytes from individuals 10 and 11 and the results show that both alleles were expressed (Figure 3a).

We investigated the methylation levels of amplicon 262 in homozygous individuals and observed a strong correlation between DNA methylation and genotype - individuals with A/A genotype were always low methylated while individuals with G/G were highly methylated (Figure 3d). The methylation levels of the A and G alleles from heterozygous individuals were similar to those of individuals with A/A or G/G genotypes, respectively (Figure 3d), suggesting that the methylation difference is determined mainly by the polymorphisms between the alleles. A similar observation was made for amplicons 23_1 and 23_2 (Additional file 6).

### Allele-specific methylation of an *RIPK4* intron

**Figure 4:**
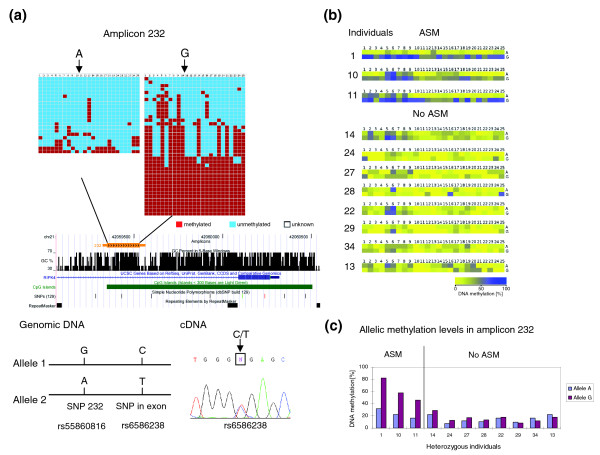
ASM of amplicon 232. (a) Example of ASM of amplicon 232. The figure shows the position of the amplicon in UCSC genome browser, the positions of the SNP (dbsnp rs55860816) used to discriminate alleles, and the allelic methylation, basically as described in the legend to Figure 2a. The correlation of the SNP in this amplicon (dbsnp rs55860816) and a SNP (rs6586238) in the first exon of the gene as identified by sequencing of genomic DNA is schematically shown in the lower part of the figure. SNP (rs6586238) was used to discriminate alleles in gene expression analysis. Sequencing of cDNA showed that the gene is biallelically expressed in leukocytes. (b) Compilation of results from all heterozygous individuals. For further details see the legend to Figure 2b. (c) Methylation level of each allele from all heterozygous individuals. The original methylation patterns of all samples with ASM are shown in Additional file 7.

Amplicon 232 is located on a CpG island in an intron of the gene *RIPK4 *near the transcriptional start site (Figure 4a). We studied the DNA methylation pattern of this region in 36 individuals. Out of the 11 individuals heterozygous at an A/G SNP in the studied region, ASM was observed in 3 cases where the A allele was always hypomethylated. In the remaining heterozygous individuals, both alleles were low methylated (Table 1; Figure 4; Additional file 7). The gene expression analysis in leukocytes from individual 10 revealed that both alleles were expressed (Figure 4a).

### Allele-specific methylation of amplicons in the *CBR1* gene

We previously identified ASM of two amplicons (176_1 and 176_2) of the *CBR1 *gene in blood derived from one individual [12]. The allele with a deletion in 176_1 (dbsnp rs41563015) and with C at a C/G SNP site in 176_2 (dbsnp rs25678) was hypermethylated when compared with the allele without deletion and with G at the SNP site. Here, we studied the methylation pattern of these two regions in 20 additional individuals (Additional file 8). For amplicon 176_1, none of these individuals had the deletion or any other SNP in the sequence and all showed low methylation. For amplicon 176_2, 7 out of 20 individuals were heterozygous, but both alleles were always unmethylated. This result suggests that the deletion was more important for ASM to occur in the *CBR1 *gene promoter than the SNP in amplicon 176_2.

## Discussion

Allele-specific methylation on autosomes is well established in imprinted genes, which carry a differentially methylated region that is methylated depending on the parental origin of the allele [4]. The sizes of such regions vary between hundreds to thousands of base pairs and the methylation difference between alleles can be up to 100%, in particular on maternally methylated alleles. Methylation leads to the exclusive expression of the maternal or paternal copy of the gene.

Recently, the allelic methylation difference of several CpG sites next to SNPs in non-imprinted regions of the genome was reported [8]. Here, we focused on whole CpG islands close to the start sites of genes and identified novel regions of ASM, which all contain a large number of CpG sites and show strong differences in average methylation of up to 85% between the alleles. In some cases the entire promoter of a gene was subject to ASM. The allele-specific differences in methylation observed here are much greater than in previously published cases.

In all cases of ASM described here, there was a full correlation between the average allelic methylation level and allelic genotype, meaning that an allele with one particular genotype was always hypermethylated in different individuals. This observation is not compatible with a connection between ASM and imprinting. We conclude that the ASM identified here is due to genetic polymorphisms between two alleles, providing examples of genotype-epigenotype interactions.

**Figure 5:**
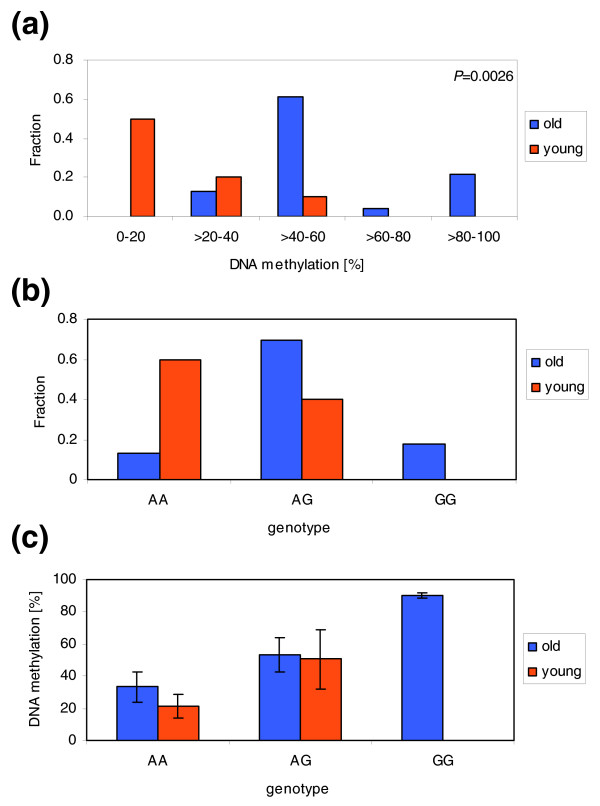
Correlation of DNA methylation with age in amplicon 262. (a) Methylation of amplicon 262 in old and young individuals analyzed without considering genotypes. An age-related highly significant increase in methylation appears to be detected. (b) Distribution of A and G genotypes among old and young individuals in our study. (c) Comparison of DNA methylation in old and young individuals with the same genotypes. This panel shows a re-analysis of the data plotted in (a); error bar represent the standard deviations of the data. This representation of the data clearly indicates that there is no age-related gain of methylation in this amplicon.

In most cases, we observed that some but not all heterozygous individuals showed ASM. Therefore, the ASM could be an outcome of an epigenetic drift, the direction of which is determined by the genetic differences between the alleles ('facilitated epigenetic variation' [13]). For one individual with ASM, we were also able to study the methylation and genotypes of the parents. In this case, the tendency to acquire hypermethylation at one allele was inherited from the mother to the son. Our data show that genetic polymorphisms strongly influence epigenetic differences among individuals, which can affect the interpretation of inter-individual variability of DNA methylation level and its potential connection to human health. For example, an initial comparison of the results obtained with amplicon 262 suggested a very significant difference between old and young individuals in the methylation levels of the right part of this amplicon (with a *P*-value of 0.0026 in two-sided *t*-test using the entire dataset; Figure 5a). However, amplicon 262 is subject to ASM and the distribution of genotypes was unequal between both groups (Figure 5b). When DNA methylation levels were compared among identical genotypes, there was no difference in the DNA methylation of old and young individuals (Figure 5c). We conclude that genotype-coupled changes in DNA methylation may influence comparative DNA methylation analyses between groups of individuals. It should be considered that (undiscovered) genetic polymorphisms outside of the region analyzed may have such effects as well.

In the current study we investigate ASM in leukocytes. We previously showed biphasic methylation of amplicon 232 in HEK293 cells, but in that sample no SNP was available to discriminate the alleles and confirm the presence of ASM (Additional file 9) [12]. We did not find evidence for ASM in any of the other amplicons in HEK293 cells, HEPG2 cells or human fibroblasts in our previous study (Additional file 9) [12]. Although the methylation status of cultured cells may not fully reflect the methylation status of the original tissue, this finding suggests that tissue specific factors play a role in the generation of ASM. There are several potential mechanisms for how genetic differences could specifically trigger the loss or gain of DNA methylation in one allele, such as binding sites of DNA binding proteins that may be present on one allele but not on the other. Predictions of transcription factor binding sites suggested for all amplicons that the different alleles might specifically interact with some transcription factors (Additional file 10), similar to what has been described recently for one case of cancer-associated ASM [9].

Our data allow for a rough estimation of how prevalent ASM is in the human epigenome. We identified 4 regions of strong ASM selected out of 190 genes studied for DNA methylation in our previous study [12]. If one considers that we found SNPs allowing us to discriminate the alleles in about 20% of all amplicons that were carefully inspected, one may estimate ASM to occur in about 10% of all genes in leukocytes. The finding that about 11% of all amplicons in different tissues showed a biphasic distribution of methylation [12] leads to a similar estimate. We conclude, therefore, that ASM likely affects many genes in the human genome.

Recently, allele-specific gene expression has been identified to be a widespread phenomenon in human cells [15-18]. The ASM may correlate with allele-specific gene expression [8,11] and we also identified one example of such correlation in our study. Aberrant monoallelic silencing of genes contributes to cancer and other diseases [19,20] and allele specific hypermethylation of genes has been associated with cancer as well [9]. Therefore, ASM may represent an important epigenetic pathway connecting genetic polymorphisms and phenotypic variability [20,21]. In the future, more genome-wide bisulfite sequencing DNA methylation analysis with DNA from different individuals will be required to uncover the full impact of genetic polymorphisms on DNA methylation and gene regulation for development and disease processes.

## Conclusions

We observed pronounced methylation difference between alleles spanning whole CpG islands in the non-imprinting loci of human autosomes, and proved a correlation with allelic gene expression level in one case. We further confirmed that the methylation differences between alleles were strongly correlated with genetic polymorphisms, which can further influence the inter-individual variability of DNA methylation. Our results suggest the ASM can affect many genes in the human genome and the genetic differences must be taken into account in future comparative DNA methylation studies.

## Materials and methods

### Genomic DNA extraction

Blood DNA was collected from 37 apparently healthy individuals, including 36 of caucasian origin, and 1 from Asia (Additional file 2). All individuals were informed about the study and declared consent in written form. Genomic DNA from blood was extracted using a QIAamp DNA Blood Mini Kit (Qiagen, Hilton, Germany).

### DNA methylation analysis

DNA methylation analysis was carried out as described [22]. Briefly, 200 to 300 ng genomic DNA was converted by bisulfite and used as template for PCR with primer designed in the region of interest. Information on amplicons selected and primer sequences are listed in Additional file 1. Around 40 clones for each amplicon were picked and sequenced using ABI BigDye Terminator chemistry (BigDye Terminator v3.1 K (Applied Biosystems Inc, Foster City, CA, USA) and 3730 × l ABI 96-capillary sequencer systems equipped with capillaries of 50-cm separation length. In all ASM cases, the presence of the SNP was confirmed by sequencing of unconverted DNA using primers listed in Additional file 1. In cases where ASM was observed, the bisulfite conversion, subcloning and sequencing analysis was performed twice in independent experiments for the same amplicon to exclude clonal PCR amplification. The final results presented here are a combination of the results from both experiments. The separate results are shown in Additional file 3.

We confirmed the methylation difference between alleles by using the methylation-sensitive restriction enzymes *Hpa*II (for amplicons 23_1, 23_2, and 232) and *Bst*UI (for amplicon 262) (New England Biolabs, Ipswich, MA, USA). Genomic DNA (250 ng) was digested at the appropriate temperature (37°C for *Hpa*II, 60°C for *Bst*UI) for 12 h in buffer recommended by the supplier. Afterwards, the PCR products were amplified from the digested genomic DNA using the following thermal cycling parameters: 15 minutes at 95°C, 35 cycles of 45 s at 95°C, 45 s at 50°C and 1 minute at 72°C, and finally 72°C for 5 minutes. The sequences of the PCR products were analyzed by direct sequencing or sequencing of subclones. The PCR products amplified from undigested genomic DNA were used as control. The primer sequences are listed in Additional file 1.

### Allele-specific mRNA expression analysis

Total RNA was extracted from leukocytes (QIAamp RNA Blood Mini Kit, Qiagen) and was reverse-transcribed. The cDNA was used as templates for PCR by using the following thermal cycling parameters: 15 minutes at 95°C, 35 cycles of 45 s at 95°C, 35 s at 55°C and 50 s at 72°C and finally 72°C for 5 minutes. The primers were designed to span different exons of genes C21orf81, *HSF2BP *and *RIPK4 *and include exonic SNPs (listed in Additional file 1). The RT-PCR product was analyzed by direct sequencing.

### Data analysis and presentation

BiQ analyzer software was used to perform quality control and to derive DNA methylation patterns from the sequencing results [23]. The BDPC (Bisulfite sequencing Data Presentation and Compilation) program was used to present the methylation pattern of single amplicons, single alleles and prepare the methylation map of all amplicons studied [24]. All data can be accessed at [25].

## Abbreviations

ASM: allele-specific methylation; CpG: cytosine-guanine dinucleotide; SNP: single nucleotide polymorphism.

## Authors' contributions

YZ and CR carried out the bisulfite conversion experiments. YZ performed all DNA methylation data analysis and gene expression analysis. CR established the website of the ASM database. RR sequenced all subclones and PCR products. CVR provided the blood samples from individuals. AJ and YZ conceived of the study, interpreted the results and wrote the paper. All authors contributed to discussion of results, and read and approved the final manuscript.

## Additional files

The following additional data are available with the online version of this paper: detailed information on the amplicons analyzed and the correlated genes, genomic positions and primers used for bisulfite genomic sequencing and gene expression analysis (Additional file 1); detailed information for all individuals selected for the analysis (Additional file 2); confirmation of the bisulfite genomic sequencing results (Additional file 3); DNA methylation patterns of amplicons 23_1 and 23_2 in different individuals (Additional file 4); DNA methylation patterns of amplicon 262 in different individuals (Additional file 5); methylation levels of homozygous individuals compared with the allelic methylation levels of heterozygous individuals for amplicons 23_1, 23_2 and 262 (Additional file 6); methylation patterns of amplicon 232 in different individuals (Additional file 7); ASM of amplicons 176_1 and 176_2 (Additional file 8); compilation of the methylation levels of regions with ASM in HEK293 cells, HEPG2 cells and human fibroblasts from [12] (Additional file 9); allele-specific transcription factor binding sites predicted at the ASM amplicons and mechanism of ASM (Additional file 10).

## Supplementary Material

Additional data file 1 Amplicons analyzed and the correlated genes, genomic positions and primers used for bisulfite genomic sequencing and gene expression analysis. Click here for file

Additional data file 2 Detailed information for all individuals selected for the analysis. Click here for file

Additional data file 3 Confirmation of the bisulfite genomic sequencing results. Click here for file

Additional data file 4 DNA methylation patterns of amplicons 23_1 and 23_2 in different individuals. Click here for file

Additional data file 5 DNA methylation patterns of amplicon 262 in different individuals. Click here for file

Additional data file 6 Methylation levels of homozygous individuals compared with the allelic methylation levels of heterozygous individuals for amplicons 23_1, 23_2 and 262. Click here for file

Additional data file 7 Methylation patterns of amplicon 232 in different individuals. Click here for file

Additional data file 8 ASM of amplicons 176_1 and 176_2. Click here for file

Additional data file 9 Methylation levels of regions with ASM in HEK293 cells, HEPG2 cells and human fibroblasts from [12]. Click here for file

Additional data file 10 Allele-specific transcription factor binding sites predicted at the ASM amplicons and mechanism of ASM. Click here for file
