# Supplementary material for: Non-imprinted allele-specific DNA methylation on human autosomes
Source: Genome Biol. 2009 Dec 3;10(12):R138. doi: 10.1186/gb-2009-10-12-r138 (PMC2812945; doi:10.1186/gb-2009-10-12-r138)
Supplement: Additional data file 1 — Amplicons analyzed and the correlated genes, genomic positions and primers used for bisulfite genomic sequencing and gene expression analysis. [file gb-2009-10-12-r138-S1.PDF]

Non-imprinted allele-specific DNA methylation on human autosomes

Yingying Zhang, Christian Rohde, Richard Reinhardt, Claudia Voelcker-Rehage & Albert Jeltsch

Additional data file 1: Detailed information of the amplicons analyzed and the correlated genes, genomic positions and primers used for bisulfite genomic sequencing.

Amplicon name	Chromosome	Strand	Start	End	Forward primer	Reverse primer
23_2	21	+	14273754	14274179	TGAGGGATGGAAAATAGTTGA	AAACAAAAACRAAAAAATAAAAA
23_1	21	-	14274136	14274505	TAGGAATTTTGAGAATGGTAAGGTT	AAAAAACAACTCCCTATCACCTTTT
140	21	-	33319383	33319703	GATGGTTTGGAGATTTTGTAGAGTT	AATCCCTACCCCCAACTAAAAA
158	21	+	34367214	34367454	TTGTTATGTTTGTTATTTAGTTTT	AAAATATTCCAAAACCCCTAC
176_1	21	+	36364032	36364333	TGTAAAGTTAGGTTAGTTGGTTTTT	ATACCCTTATTACCTCCAATCACC
176_2	21	+	36364230	36364705	GGGTTTGAGTTAGGTTTGTTTTTTA	TCCAAAATTTCTACACTCCCTACAT
187	21	+	36774786	36775091	GGTTTGGGGATTTTTTTAAGGTATA	AACACAAAAACCAAACTTTTAATCC
197	21	-	37562188	37562464	TTGGGAATAAAAATAATAGGTAGATTTATT	CAACTAAACCTAAAACCAAAATCTC
223	21	+	41140973	41141244	GAGTAGGGATTTTTTTTTTGTT	RAACATTTCATATTCACCTCTC
229	21	-	41719790	41720129	GTTTTTTTAATGTTTTTTTT	AAACACCTTAATCCTCAAAC
232	21	+	42059323	42059563	GAATTTGAATGTAAGGTTTTTTTT	AACACAAATTAATATAAAACTATAATTATT
257	21	+	43461221	43461648	GGGAGTGTTTGGATATTTAGTGAGA	TTCCAACACCTAAAAAAACCCTATA
262	21	-	43902246	43902635	TTGTAGATAATAAGTTTAAAATT	AATCTCCAAACTTCCCCCAAA
283	21	+	44700205	44700569	TTTTTTTAGGTAGTTGAAAGAAAAGG	CATAACCCAATAAATAATCACTAC
307	21	+	45165207	45165481	GGGGGTTTTGTTATTAGTTTGTTTT	AACCCCTCTCCCAAATATAAATCTA
335	21	+	46849231	46849623	GTTGTTAATTGTTTTAAAGGGGTTTT	TCCTCAACCCATATACAATCCTAAC

Amplicon name	gene name	Description	Length (bp)	CpG sites
23_1	C21orf81	chromosome 21 open reading frame 81	370	31
23_2	C21orf81	chromosome 21 open reading frame 81	426	26
140	OLIG2	oligodendrocyte lineage transcription factor 2	321	20
158	MRPS6	mitochondrial ribosomal protein S6	241	31
176_1	CBR1	carbonyl reductase 1	302	31
176_2	CBR1	carbonyl reductase 1	476	44
187	CLDN14	claudin 14	241	10
197	DSCR3	Down syndrome critical region gene 3	272	22
223	DSCAM	Down syndrome cell adhesion molecule	321	12
229	MX1	myxovirus (influenza virus) resistance 1, interferon-inducible protein p78 (mouse)	428	33
232	RIPK4	receptor-interacting serine-threonine kinase 4	241	25
257	CRYAA	crystallin, alpha A	241	20
262	HSF2BP	heat shock transcription factor 2 binding protein	400	42
283	LRRC3	leucine rich repeat containing 3	306	28
307	ITGB2	integrin, beta 2 (complement component 3 receptor 3 and 4 subunit)	275	9
335	S100B	S100 calcium binding protein, beta (neural)	277	10

Primers used for genomic DNA sequencing to identify the genotype and confirm the methylation difference between alleles.

Amplicon	Forward Primer	Reverse Primer
23_1/23_2	GGTGGTGACTTCTCAGACTC	GATCCGTGGCTGCACTATTC
262	GCCGTTAGGGGAGGAAGTCT	AAATTCTGGGAGGTTTGGGC
232_A	TTCAAGAACATTCTCCCTACC	AAGAAAAGCAGGGCAGCTGAT
232_B	TGACTTTTGGACTGGGAAAC	TACGTTGGAACACAAATTGG

Primers for 23_1/23_1 and 262 were used both for the identification of genotype and

confirmation of the methylation difference between alleles. Primers 232_A were used to identify the genotype of gene RIPK4. Primers 232_B were used to amplify the PCR products from genomic DNA with or without methylation sensitive restriction enzymes digestion.

Primers for RT-PCR

Amplicon	Forward Primer	Reverse Primer	SNPs in exon
23_1/23_2	TGGGGTTGGATAGGGGATTT	CACTTTCTTGGGGACACACT	rs56270809
262	CTCTCTAGGCCGAGAATACTGC	TTCCAGATCCTTCTTTCTGACTTTA	rs2838343
232	GGTGCGCCATGTCCACTGGAAG	CCGGAGATCCCATGGCAATGGC	rs6586238

For amplicon 23_1/23_2 and 262, the SNPs analyzed in the amplicons are located in the exons of the genes, such that the SNPs were directly used to discriminate the alleles in gene expression analysis. For amplicon 232, SNP rs6586238 in the first exon of the gene was used to discriminate alleles and its correlation with the SNP rs55860816 in amplicon 232 was identified in genomic DNA.
