# Supplementary material for: Non-imprinted allele-specific DNA methylation on human autosomes
Source: Genome Biol. 2009 Dec 3;10(12):R138. doi: 10.1186/gb-2009-10-12-r138 (PMC2812945; doi:10.1186/gb-2009-10-12-r138)
Supplement: Additional data file 2 — Detailed information for all individuals selected for the analysis. [file gb-2009-10-12-r138-S2.PDF]

Non-imprinted allele-specific DNA methylation on human autosomes

Yingying Zhang, Christian Rohde, Richard Reinhardt, Claudia Voelcker-Rehage & Albert Jeltsch

Additional data file 2: Detailed information for all individuals selected for the analysis.

Individual	Age	Gender	origin
1	75	male	caucasian
2	69	male	caucasian
3	67	male	caucasian
4	72	male	caucasian
5	64	male	caucasian
6	71	female	caucasian
7	73	female	caucasian
8	71	female	caucasian
9	71	female	caucasian
10	65	female	caucasian
11	21	female	caucasian
12	25	female	caucasian
13	28	female	caucasian
14	30	female	caucasian
15	27	female	caucasian
16	26	male	asian
17	27	male	caucasian
18	32	male	caucasian
19	22	male	caucasian

Individual	Age	Gender	origin
20	26	male	caucasian
21	68	female	caucasian
22	74	female	caucasian
23	66	female	caucasian
24	71	female	caucasian
25	74	female	caucasian
26	65	female	caucasian
27	64	female	caucasian
28	62	female	caucasian
29	66	female	caucasian
30	69	male	caucasian
31	79	male	caucasian
32	72	male	caucasian
33	68	male	caucasian
34	67	male	caucasian
35	69	male	caucasian
17_P	63	male	caucasian
17_M	57	female	caucasian

17_P: father of individual 17. 17_M: mother of individual 17.

The individual analyzed previously in NAME21 project (12) was labeled as N in the text and figures.
