# Supplementary material for: Non-imprinted allele-specific DNA methylation on human autosomes
Source: Genome Biol. 2009 Dec 3;10(12):R138. doi: 10.1186/gb-2009-10-12-r138 (PMC2812945; doi:10.1186/gb-2009-10-12-r138)

Non-imprinted allele-specific DNA methylation on human autosomes

Yingying Zhang, Christian Rohde, Richard Reinhardt, Claudia Voelcker-Rehage & Albert Jeltsch

Additional data file 3: Confirmation of the bisulfite genomic sequencing results

A) To confirm the ASM identified by the bisulfite genomic sequencing, individual bisulfite genomic sequencing analyses were performed twice for each amplicon, with allele-specific methylation difference. Identical results were observed in all repeats, examples of which are shown in the figure. This result shows that clonal PCR amplification cannot be the reason for the allelic methylation differences.

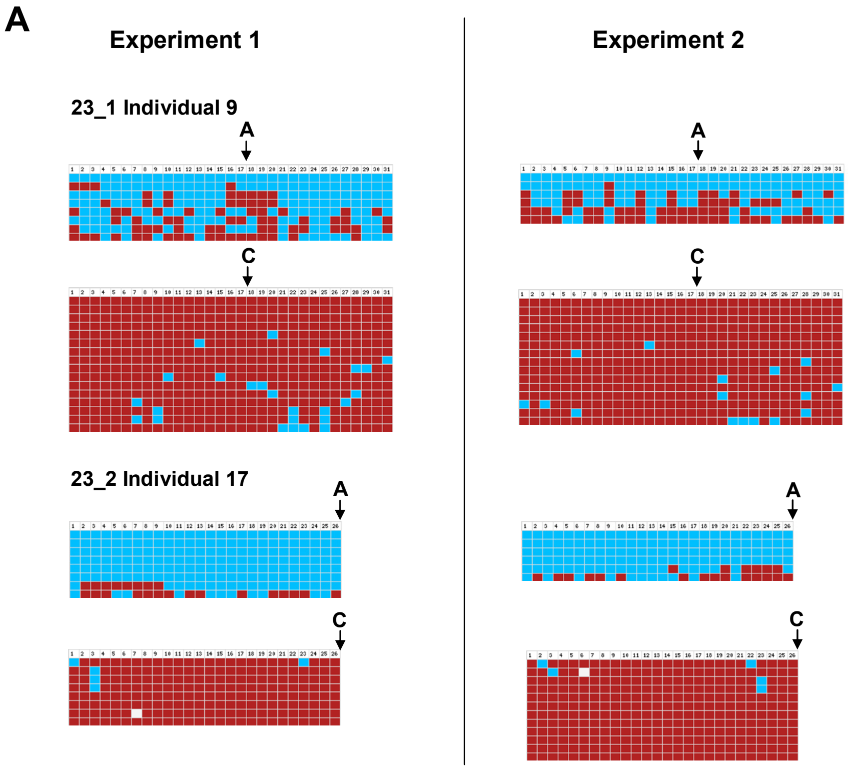

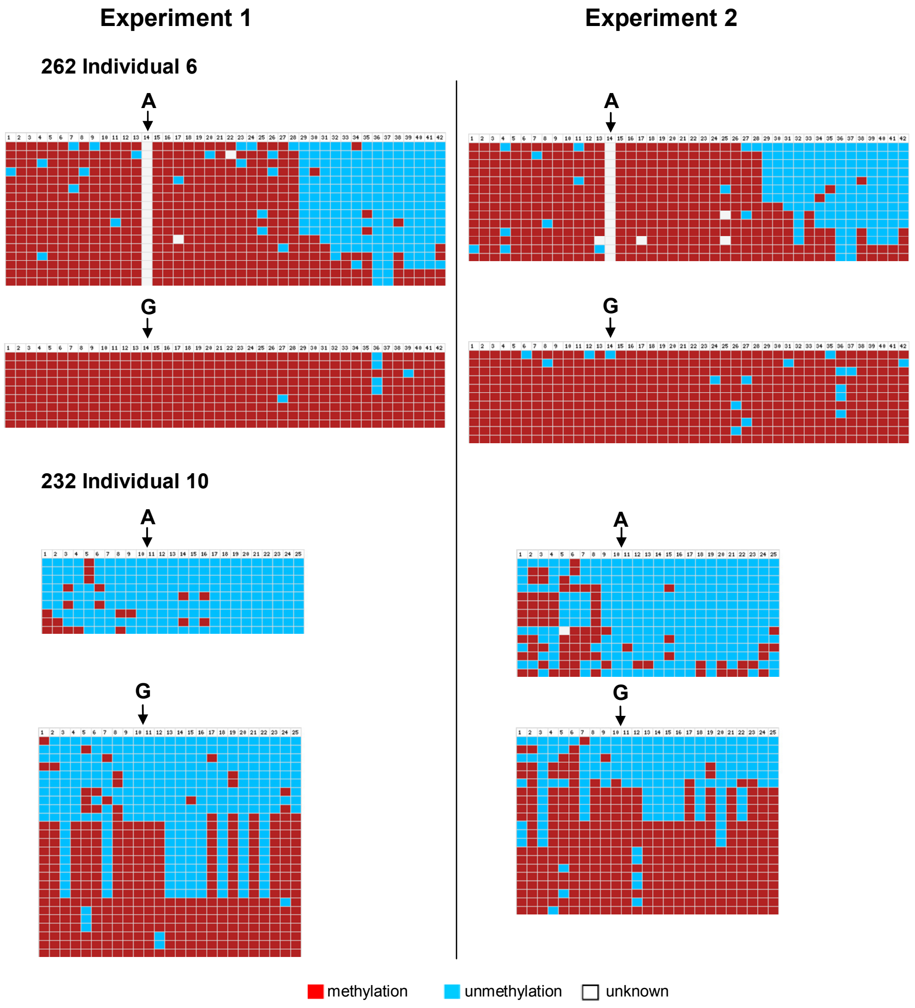

B) We confirmed the methylation difference between alleles by digesting the genomic DNA with methylation sensitive restriction enzymes HpaII and BstUI. HpaII has 3 cleavage sites in an amplicon covering 23_1 and 23_2, and 5 cleavage sites in amplicon 232. BstUI has 3 cleavage sites in amplicon 262, one of which is located in the differentially methylated part of the amplicon. This was followed by PCR amplification of the ASM regions and sequencing for the amplicons 232 and 262. For amplicons 23_1 and 23_2 direct sequencing of the PCR product technically failed. Therefore the PCR product was subcloned and individual clones were sequenced. As shown in the figure, digestion with restriction enzymes in all cases led to an enrichment of the methylated allele in the PCR product when compared to the genomic DNA without digestion (the C-allele for amplicon 23_1 and 23_2, the

G-alleles for amplicon 262 and 232). This result confirms the allele-specific methylation observed in the bisulfite genomic sequencing data.

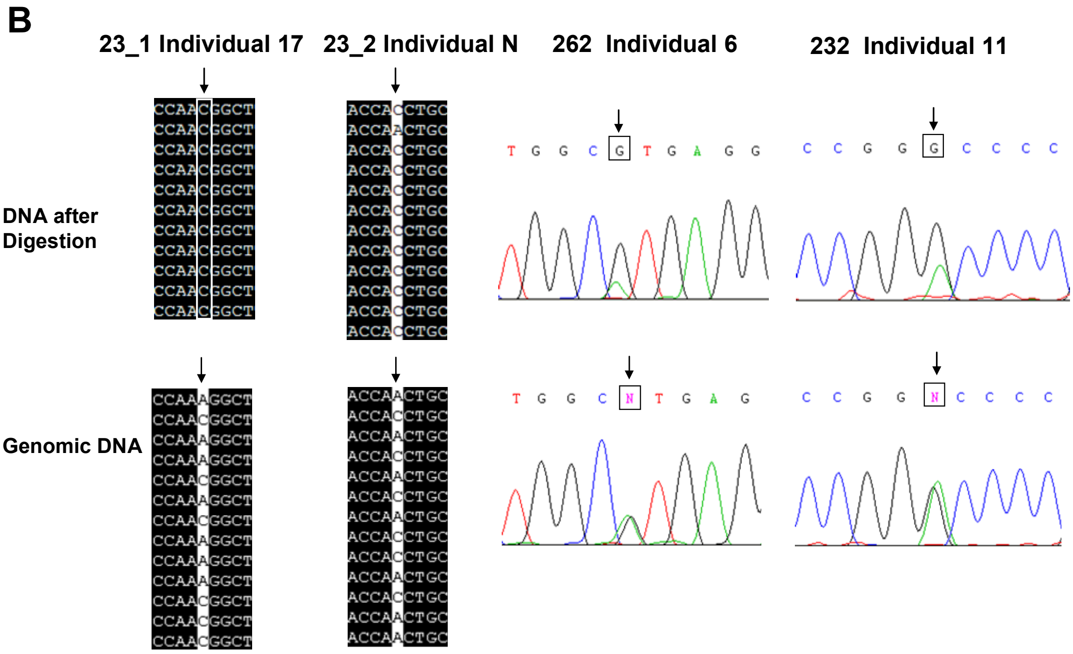

Supplement: Additional data file 3 — Confirmation of the bisulfite genomic sequencing results. [file gb-2009-10-12-r138-S3.PDF]
