# Supplementary material for: Non-imprinted allele-specific DNA methylation on human autosomes
Source: Genome Biol. 2009 Dec 3;10(12):R138. doi: 10.1186/gb-2009-10-12-r138 (PMC2812945; doi:10.1186/gb-2009-10-12-r138)

Non-imprinted allele-specific DNA methylation on human autosomes

Yingying Zhang, Christian Rohde, Richard Reinhardt, Claudia Voelcker-Rehage & Albert Jeltsch

Additional data file 4: DNA methylation patterns of amplicon 23_1 and 23_2 in different individuals

In all figures, the subclones of every PCR product were sorted according to the presence of polymorphisms in the sequence. The positions of the SNPs are indicated by arrows. Each row corresponds to one subclone, each column corresponds to one CpG site in the studied region. The color code indicates different methylation states of the CpG sites (blue: unmethylated, red: methylated, white: methylation state unknown, usually because of ambiguous sequencing results).

1. Methylation pattern of amplicon 23_1 in different individuals

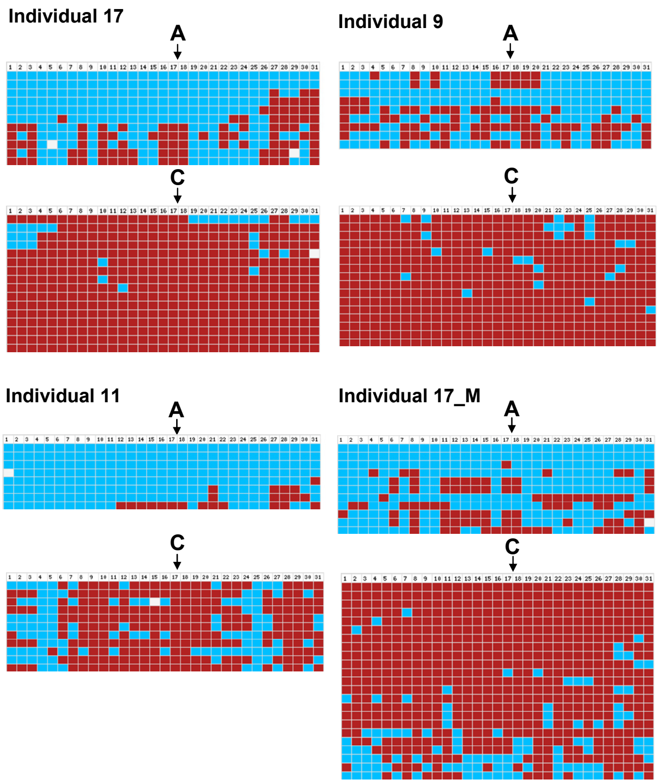

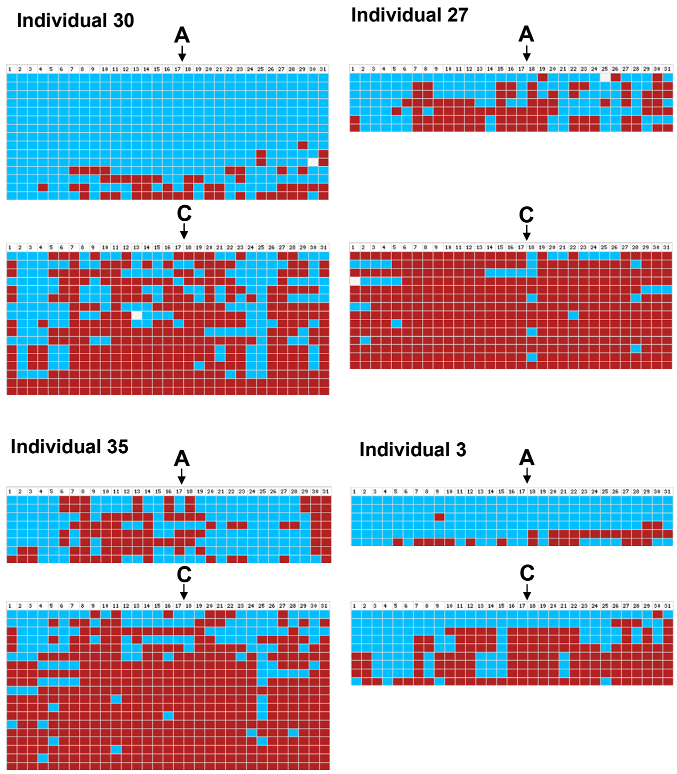

2. Methylation pattern of amplicon 23_1 in the parents of individual 17

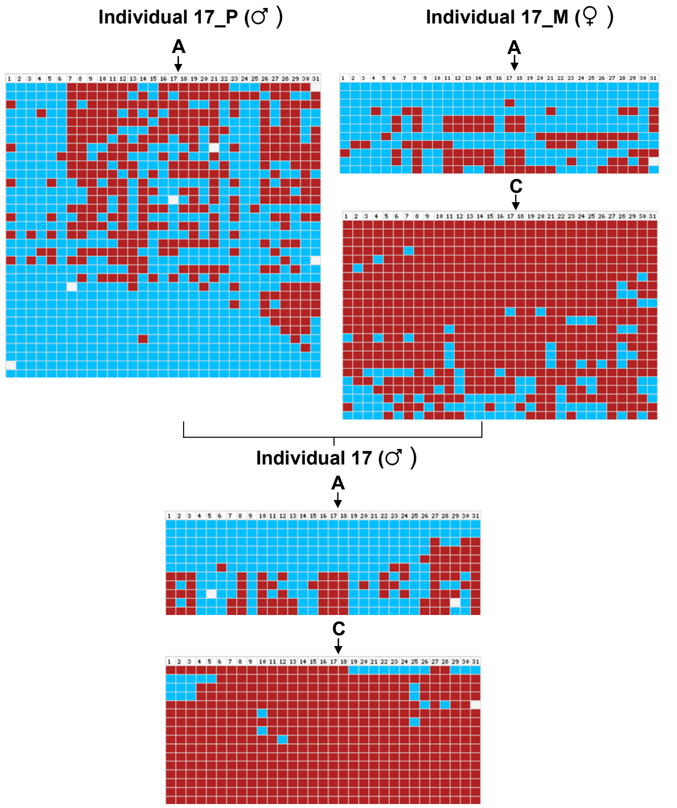

3. Methylation pattern of amplicon 23_2 in different individuals

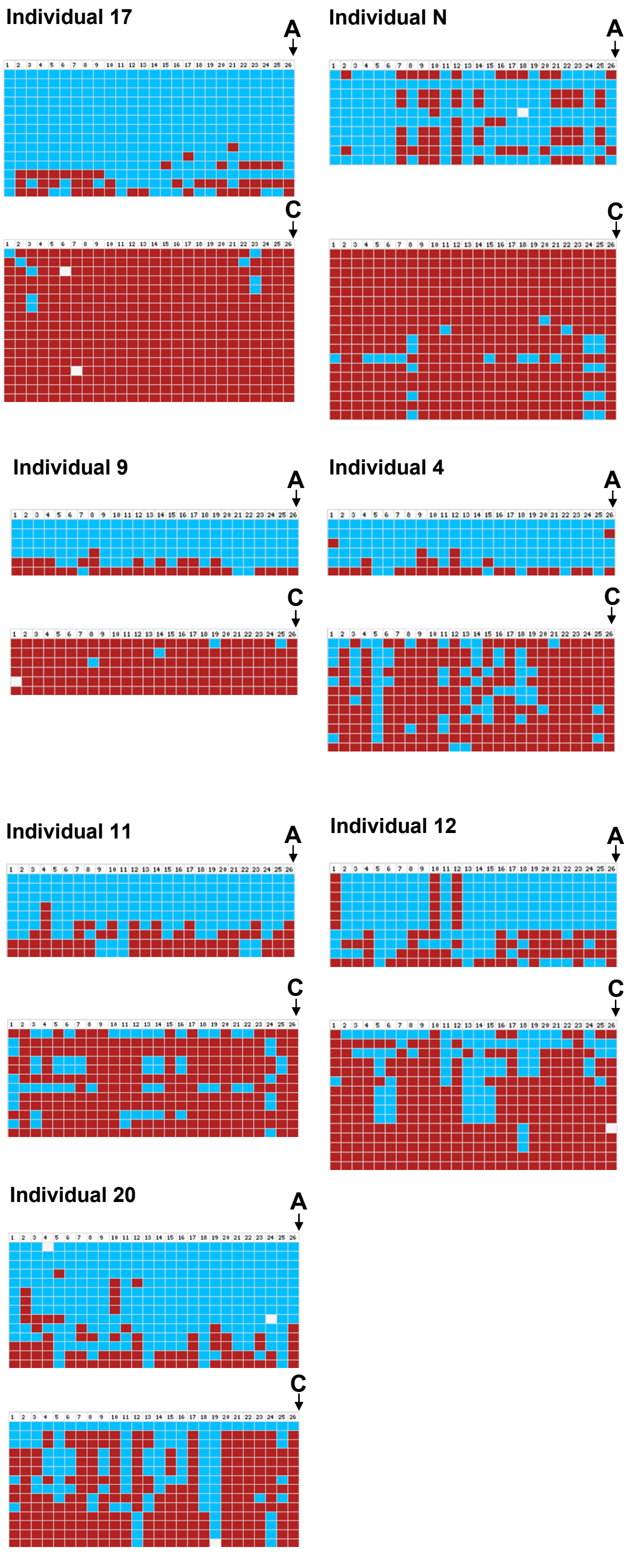

4. Correlation of methylation patterns of amplicon 23_1 and 23_2.

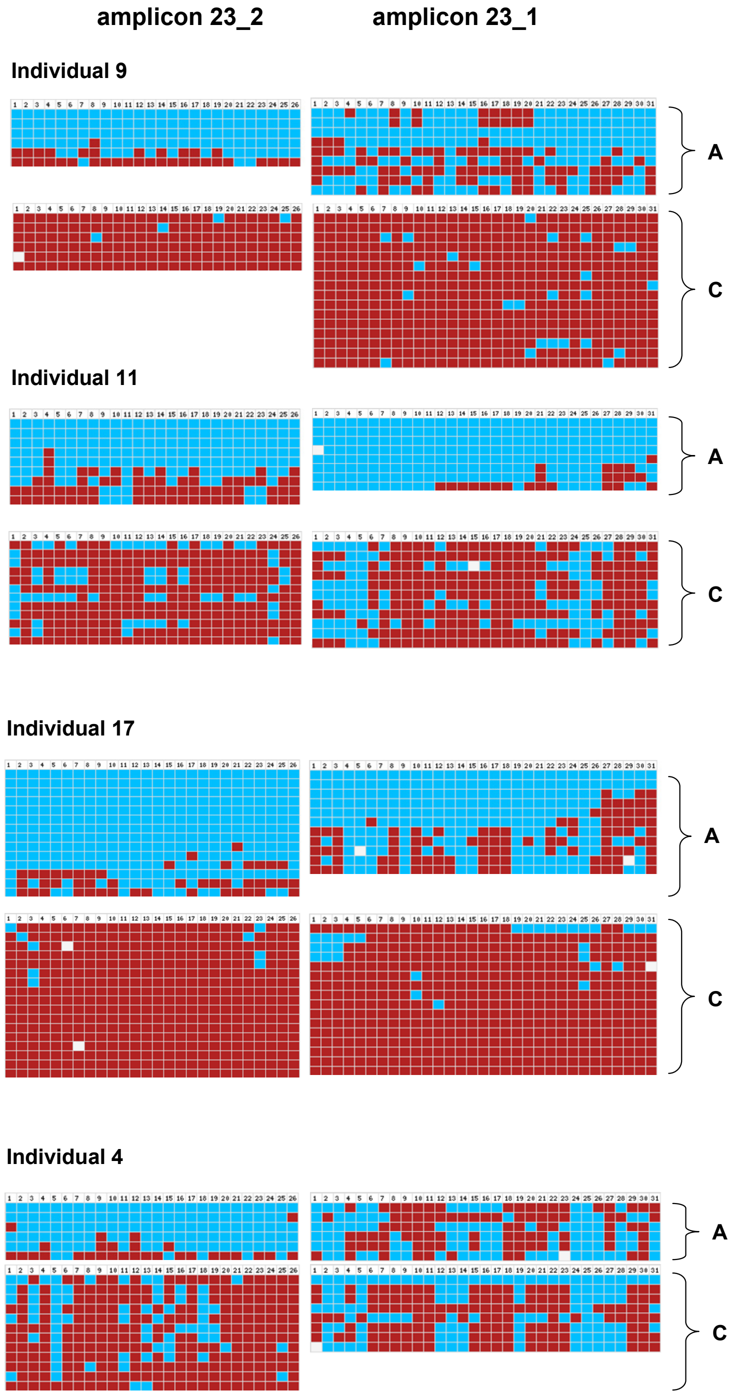

Individual 12

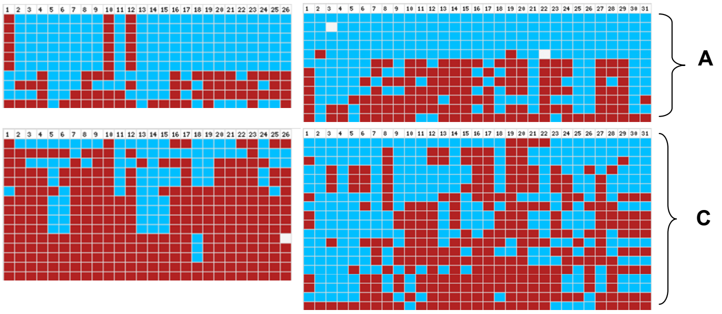

Individual 20

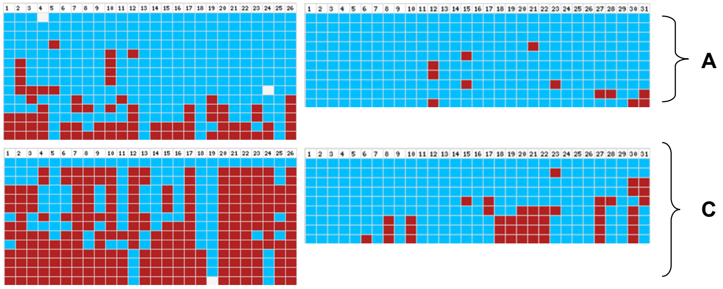

Individual 1

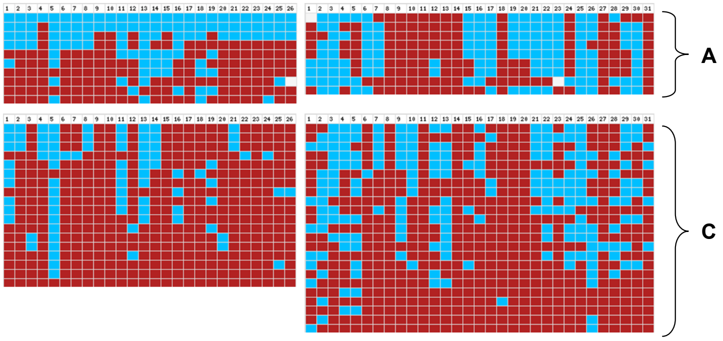

Supplement: Additional data file 4 — DNA methylation patterns of amplicons 23_1 and 23_2 in different individuals. [file gb-2009-10-12-r138-S4.PDF]
