# Supplementary material for: Non-imprinted allele-specific DNA methylation on human autosomes
Source: Genome Biol. 2009 Dec 3;10(12):R138. doi: 10.1186/gb-2009-10-12-r138 (PMC2812945; doi:10.1186/gb-2009-10-12-r138)
Supplement: Additional data file 5 — DNA methylation patterns of amplicon 262 in different individuals. [file gb-2009-10-12-r138-S5.PDF]

Non-imprinted allele-specific DNA methylation on human autosomes

Yingying Zhang, Christian Rohde, Richard Reinhardt, Claudia Voelcker-Rehage & Albert Jeltsch

Additional data file 5: Methylation pattern of amplicon 262 in different individuals.

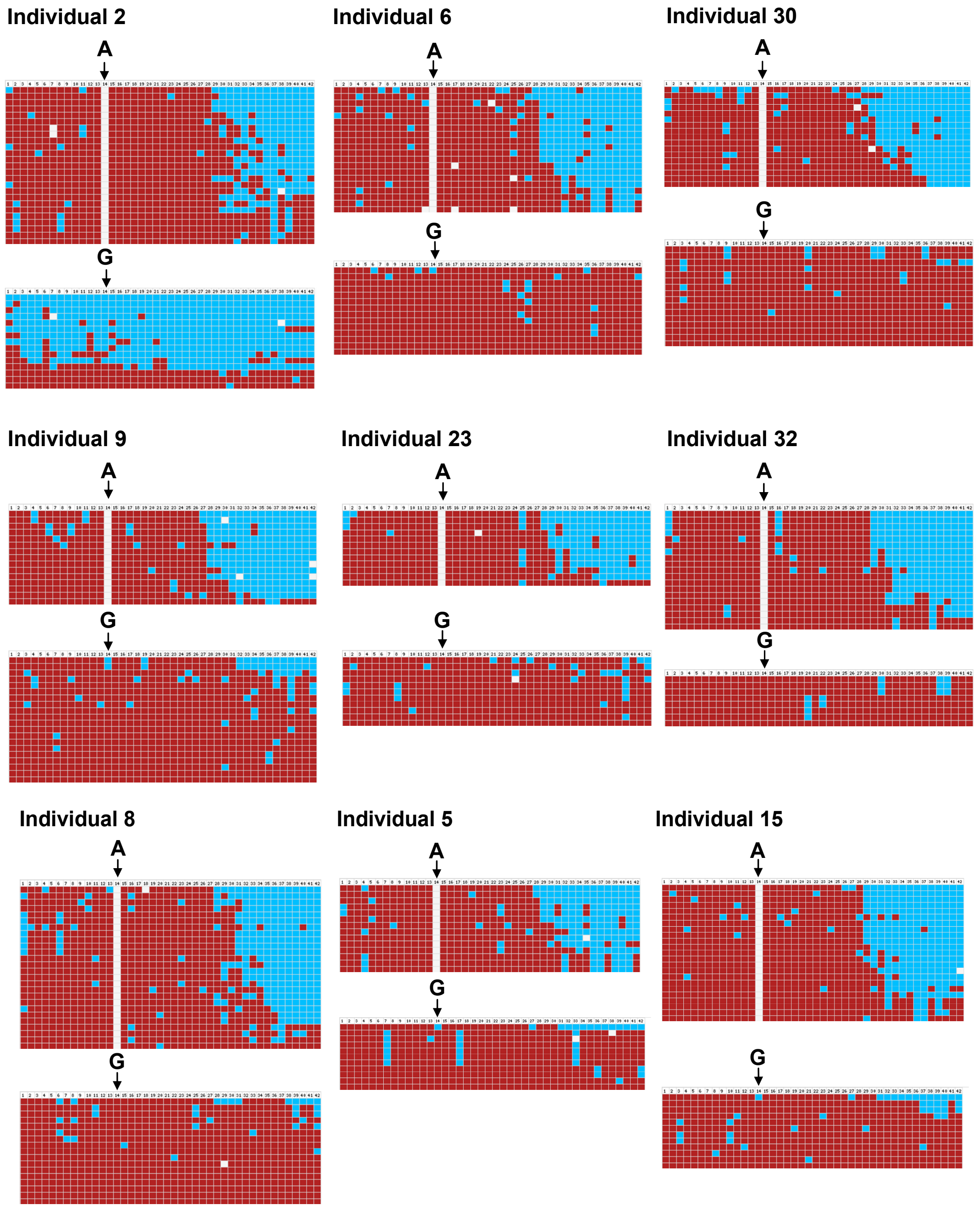

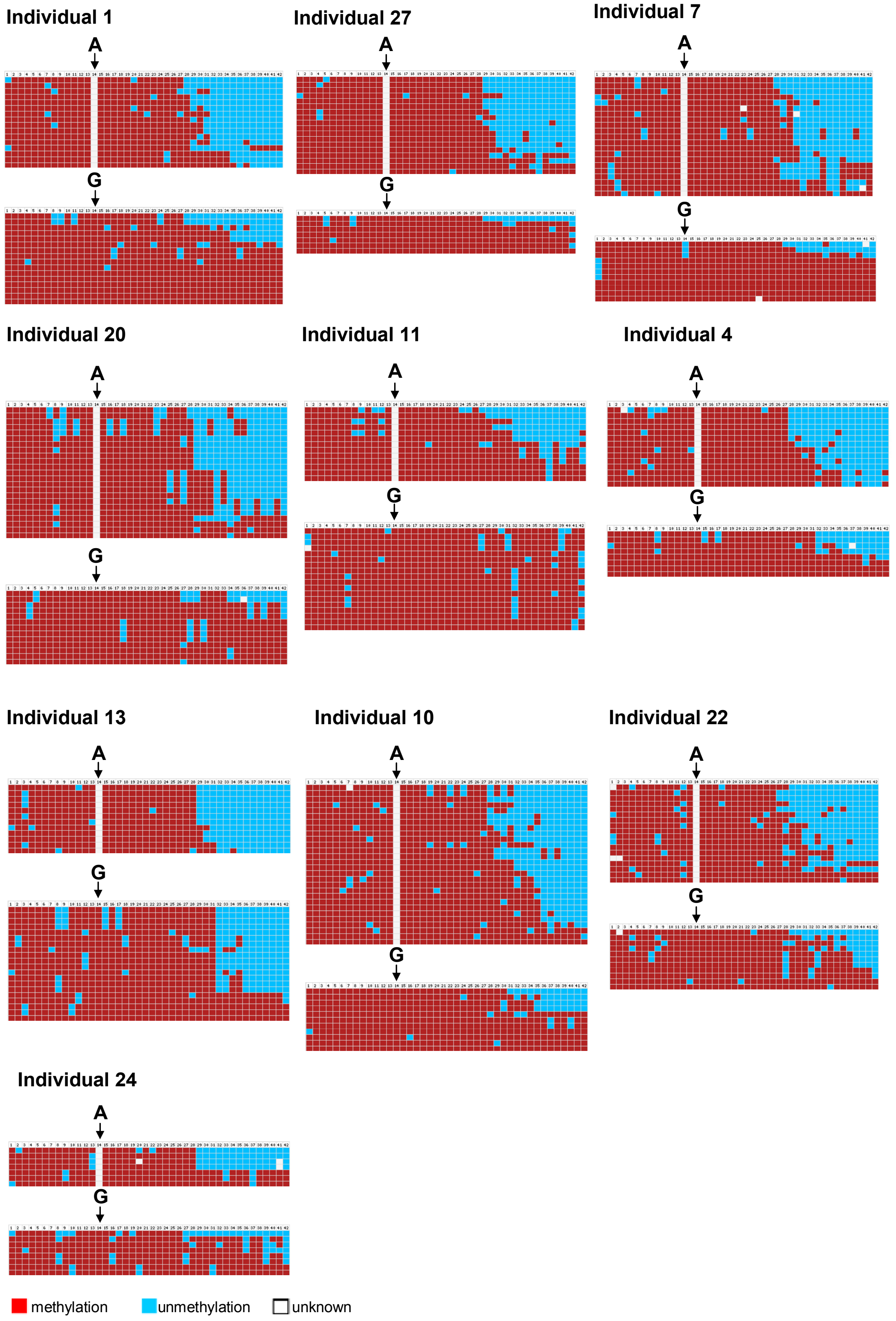
Individual 1
Individual 27
Individual 7
Individual 20
Individual 11
Individual 4
Individual 13
Individual 10
Individual 22
Individual 24
A
G
methylation
unmethylation
unknown
