# Supplementary material for: Non-imprinted allele-specific DNA methylation on human autosomes
Source: Genome Biol. 2009 Dec 3;10(12):R138. doi: 10.1186/gb-2009-10-12-r138 (PMC2812945; doi:10.1186/gb-2009-10-12-r138)

Non-imprinted allele-specific DNA methylation on human autosomes

Yingying Zhang, Christian Rohde, Richard Reinhardt, Claudia Voelcker-Rehage & Albert Jeltsch

Additional data file 6: Methylation levels of homozygous individuals compared with the allelic methylation levels of heterozygous individuals for the amplicons 23_1, 23_2 and 262. The graphs display the cumulative fraction of individuals for each DNA methylation level. The numbers of individuals with corresponding genotypes (N) are specified in the legends.

Amplicon 23_1

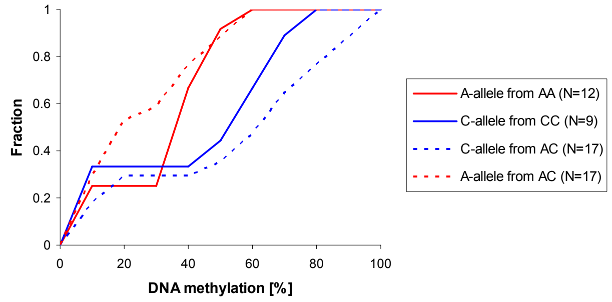

Amplicon 23_2

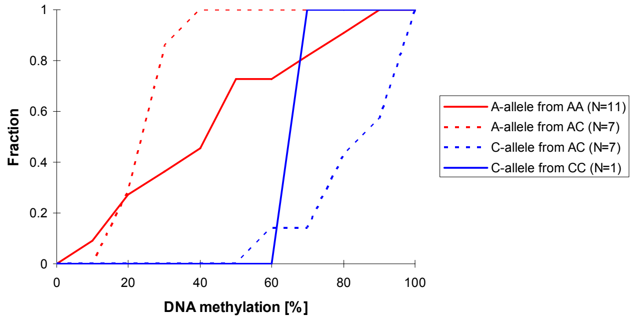

Amplicon 262

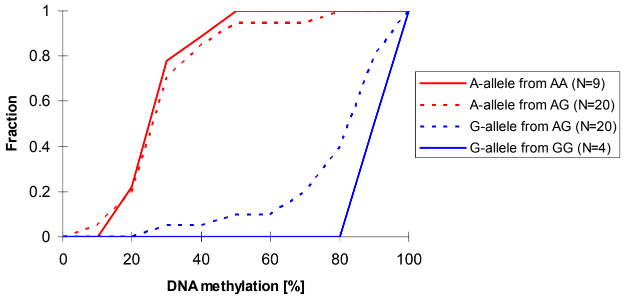

Supplement: Additional data file 6 — Methylation levels of homozygous individuals compared with the allelic methylation levels of heterozygous individuals for amplicons 23_1, 23_2 and 262. [file gb-2009-10-12-r138-S6.PDF]
