# Supplementary material for: Non-imprinted allele-specific DNA methylation on human autosomes
Source: Genome Biol. 2009 Dec 3;10(12):R138. doi: 10.1186/gb-2009-10-12-r138 (PMC2812945; doi:10.1186/gb-2009-10-12-r138)

Non-imprinted allele-specific DNA methylation on human autosomes

Yingying Zhang, Christian Rohde, Richard Reinhardt, Claudia Voelcker-Rehage & Albert Jeltsch

Additional data file 8: ASM of amplicons 176_1 and 176_2.

A) ASM of amplicon 176_1 and 176_2 as observed in individual N (12). The figure shows the position of the two amplicons in UCSC genome browser, the positions of the deletion (dbsnp rs41563015) and SNP (dbsnp rs25678) used to discriminate alleles and the allelic methylation. By sequencing of the genomic DNA we identified that the deletion correlates with C-allele at the SNP site as schematically shown in the lower part of the figure.

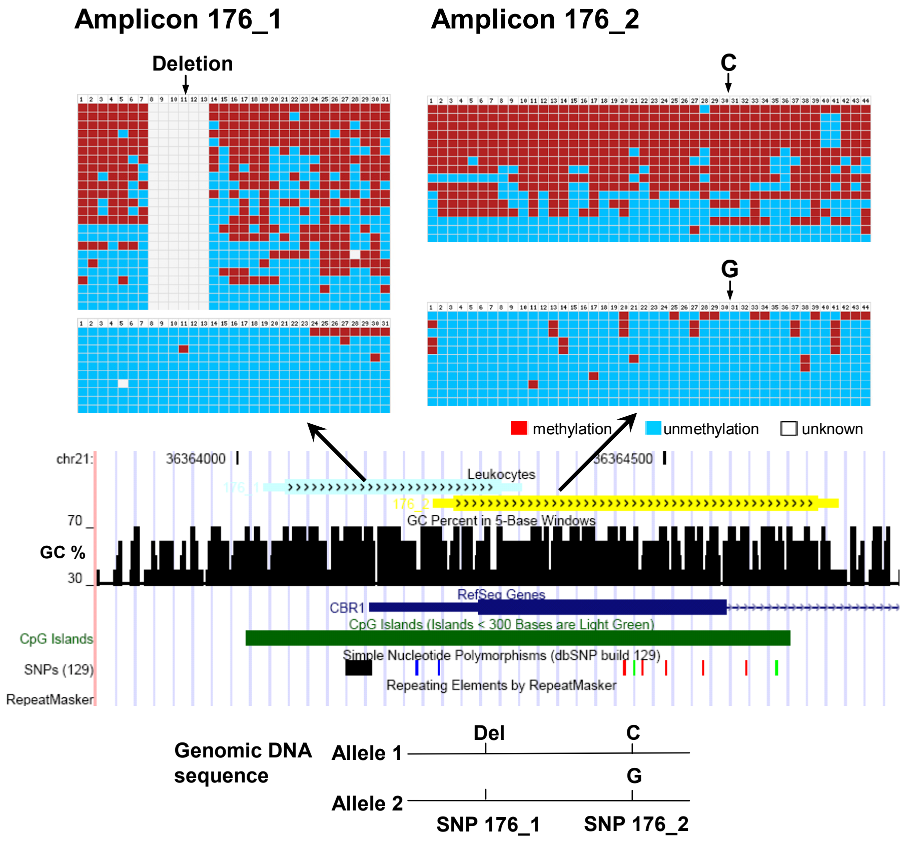

B) Upper part: DNA methylation level of amplicon 176_1 in both alleles of individual N (12). Lower part: methylation levels of amplicon 176_1 in 20 individuals which did not have a genetic difference in this amplicon.

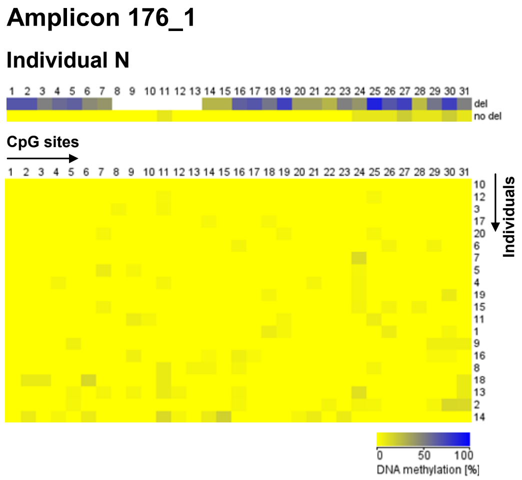

C) Upper part: DNA methylation level of amplicon 176_2 in both alleles of individual N (12). Lower part: methylation levels of amplicon 176_2 in 7 individuals with SNP.

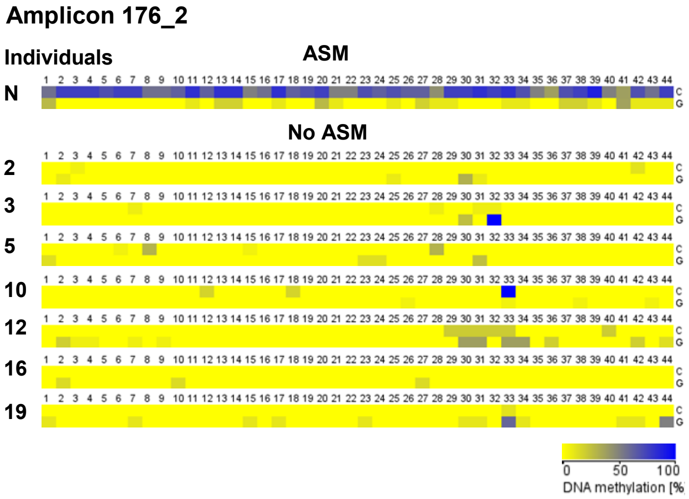

Supplement: Additional data file 8 — ASM of amplicons 176_1 and 176_2. [file gb-2009-10-12-r138-S8.PDF]
