# Supplementary material for: Non-imprinted allele-specific DNA methylation on human autosomes
Source: Genome Biol. 2009 Dec 3;10(12):R138. doi: 10.1186/gb-2009-10-12-r138 (PMC2812945; doi:10.1186/gb-2009-10-12-r138)
Supplement: Additional data file 9 — Methylation levels of regions with ASM in HEK293 cells, HEPG2 cells and human fibroblasts from [12]. [file gb-2009-10-12-r138-S9.PDF]

Non-imprinted allele-specific DNA methylation on human autosomes

Yingying Zhang, Christian Rohde, Richard Reinhardt, Claudia Voelcker-Rehage & Albert Jeltsch

Additional data file 9: Compilation of the methylation level of regions with ASM in HEK293 cells, HEPG2 cells and human fibroblasts (12).

Amplicon name	cell type		
	HEK293	HEPG2	Fibroblasts
23_1	unmethylated<br>not biphasic	73% methylated<br>not biphasic	54% methylated<br>not biphasic
23_2	unmethylated<br>not biphasic	81% methylated<br>not biphasic	77% methylated<br>not biphasic
262	96% methylated<br>not biphasic	97% methylated<br>not biphasic	96% methylated<br>not biphasic
232	83% methylated<br>biphasic, no SNP	unmethylated<br>not biphasic	67% methylated<br>not biphasic

The amplicons 23_1, 23_2, 262 and 232 of this study correspond to the amplicons 23, 23_2, 262, 232new_1 in the previous work (12).
