# Supplementary material for: Non-imprinted allele-specific DNA methylation on human autosomes
Source: Genome Biol. 2009 Dec 3;10(12):R138. doi: 10.1186/gb-2009-10-12-r138 (PMC2812945; doi:10.1186/gb-2009-10-12-r138)
Supplement: Additional data file 10 — Allele-specific transcription factor binding sites predicted at the ASM amplicons and mechanism of ASM. [file gb-2009-10-12-r138-S10.PDF]

Non-imprinted allele-specific DNA methylation on human autosomes

Yingying Zhang, Christian Rohde, Richard Reinhardt, Claudia Voelcker-Rehage & Albert Jeltsch

Additional data file 10: Allele-specific TF binding sites predicted at the ASM amplicons and mechanism of ASM

The presence of an Sp1/Sp3 binding site has been correlated to cancer related ASM (9). Predictions of TF binding site using TFSEARCH program (26) suggest for all amplicons described here, that the different alleles might specifically bind different transcription factors. The deleted region in 176_1 is predicted to contain an SP1 binding site. In amplicon 23_2, the methylated allele is predicted to bind USF and E47, while the unmethylated allele could bind Myb. In amplicon 23_1, the methylated allele could specifically bind Myb at the SNP site. In amplicon 232, p300 is predicted to specially bind to the methylated allele. In amplicon 262, the unmethylated allele could specifically bind Aml-1a. The differential binding of transcription factors to alleles could affect the methylation states of alleles by recruiting transcriptional co-activators or co-repressors. Alternatively, repeats present in one allele outside of to the regions analyzed might attract the methylation and/or mutations may have generated or disrupted insulators between constitutively methylated regions and the ASM regions.
